# Yirui Capsules Alleviate Atherosclerosis by Improving the Lipid Profile and Reducing Inflammation in Apolipoprotein E-Deficient Mice

**DOI:** 10.3390/nu10020142

**Published:** 2018-01-29

**Authors:** Jiqu Xu, Zumeng Xia, Shuang Rong, Hui Gao, Wei Yang, Jieliang Li, Congcong Ma, Qianchun Deng, Qingde Huang, Lingyun Xiao, Fenghong Huang

**Affiliations:** 1 Department of Nutriology, Oil Crops Research Institute, Chinese Academy of Agricultural Sciences, Wuhan 430062, China; xujiqu@caas.cn (J.X.); ccongma@163.com (C.M.); qchun2@126.com (Q.D.); viperson1973@163.com (Q.H.); 2 Hubei Key Laboratory of Lipid Chemistry and Nutrition, Oil Crops Research Institute, Chinese Academy of Agricultural Sciences, Wuhan 430062, China; 3 Key Laboratory of Oilseeds processing, Ministry of Agriculture, Wuhan 430062, China; 4 Functional Oil Laboratory Associated by Oil Crops Research Institute, Chinese Academy of Agricultural Sciences and Infinitus (China) Company Ltd., Guangzhou 510663, China; Xiaolingyun12@126.com; 5 Department of Nutrition and Food Hygiene, School of Public Health, Medical College, Wuhan University of Science and Technology, Wuhan 430065, China; shuang_rong@yeah.net; 6 Department of Nutrition and Food Hygiene, School of Public Health, Tongji Medical College, Huazhong University of Science and Technology, Wuhan 430030, China; gaohuitj@yeah.net (H.G.); yangw1983@foxmail.com (W.Y.); 7 Department of Pathology and Laboratory Medicine, Temple University School of Medicine, Philadelphia, PA 19140, USA; Jieliang.li@temple.edu

**Keywords:** Yirui capsule, atherosclerosis, lipid profile, inflammation

## Abstract

Atherosclerosis (AS) is the main cause of cardiovascular diseases. This study investigated Yirui (YR) capsules, whose ingredients are available in health food stores, against AS and the underlying mechanisms. Male apolipoprotein E-deficient mice fed a high-fat diet for 10 weeks developed severe aortic lesions, but YR significantly decreased the plaque area in the total aorta and aortic root. YR affected the serum lipid profile by significantly reducing total cholesterol, low-density lipoprotein cholesterol (LDL-C), triglyceride (TG), and oxidative modification of LDL-C (Ox-LDL) levels. In addition, multi-cytokine analysis revealed that higher serum levels of interleukin-1 alpha (IL-1α), interleukin-1 beta (IL-1β), interleukin-3 (IL-3), interleukin-6 (IL-6), interleukin-27 (IL-27), tumor necrosis factor alpha, interferon gamma, and regulated on activation, normal T cell expressed and secreted (RANTES), which were induced by a high-fat diet, declined with YR treatment. These results suggest that YR reduces the atherosclerotic plaque burden, thereby alleviating AS by modulating the lipid profile and inhibiting inflammation.

## 1. Introduction

Cardiovascular diseases (CVDs), which represent 31% of all global deaths, continue to be the leading cause of death worldwide [1]. Atherosclerosis (AS) is the most common pathological process that leads to CVD and is characterized by the accumulation of lipids and fibrous elements in the large arteries.

Abundant data have linked dyslipidemia and chronic low-grade inflammation to AS. Hyperlipidemia is serologically characterized by increased plasma triglyceride (TG), total cholesterol (TC), and low-density lipoprotein cholesterol (LDL-C) levels [2]. Since hypercholesterolemia leads to cholesterol accumulation in the artery wall, which initiates the development of AS [3], it may be unique in being sufficient to cause the development of atherosclerotic lesions, even in the absence of other cardiovascular risk factors [4]. Elevated circulating LDL-C levels are a well-established major risk factor for AS, as supported by clinical evidence showing decreased atherosclerotic disease events when LDL-C was therapeutically lowered. The oxidative modification of LDL-C (Ox-LDL) is a crucial step in the development of AS [4,5,6] because it directly drives the progression of AS in all stages of this disease by many mechanisms [4,7,8,9] and is a biomarker of CVD [10]. Hypertriglyceridemia may also be a significant cardiovascular risk factor [11,12]. AS is a chronic inflammatory and immunological disease involving many cell types, including monocytes and macrophages. Many risk factors, such as dyslipidemia, hypertension, and obesity, trigger multiple inflammatory reactions that lead to monocyte recruitment and foam cell formation in AS lesions [13]. For example, LDL elicits vascular inflammation which drives the build-up of lipid-laden atherosclerotic plaques [14]. The interplay between lipid metabolism and inflammation aggravates the development of AS [15].

Traditional Chinese Medicine (TCM) has been extensively used in China for thousands of years and is seen as a valuable asset for health promotion. Some TCM formulas have anti-atherosclerotic properties [16,17]. Yirui (YR) capsules are an innovative TCM formula, which were designed to improve hyperlipidemia. Its major ingredients include *Radix Salviae miltiorrhizae* (Danshen), *Fructus Crataegi* (Shanzha), *Rhizoma Alismatis* (Zexie), perilla oil, *Radix Notoginseng* (Sanqi), and *Folium Ginkgo* (Yinxingye), all of which are Chinese health food ingredients [18]. The ingredients are beneficial for the prevention or treatment of CVDs by many mechanisms such as blood lipid regulation [19,20,21,22,23,24], anti-oxidation [23,25,26], and anti-inflammation [23,25,26,27,28]. Indeed, YR has been shown to improve serum lipid profiles and the inflammatory response in hyperlipidemic rat models [29]. Therefore, in this study, we determined whether this formula has beneficial effects on the progression of AS.

## 2. Materials and Methods

### 2.1. Animals

Male apolipoprotein E-deficient (ApoE^−/−^) mice in a C57BL/6J background and wild-type (C57BL/6J) male mice were purchased from the Vital River Laboratory Animal Center (Beijing, China) at seven weeks of age. The animals were housed individually and maintained at a controlled ambient temperature (22 ± 1 °C) under diurnal conditions (light–dark: 08:00–20:00) with access to standard laboratory rodent chow and tap water *ad libitum*. After acclimatization for seven days, C57BL/6J mice were given a low fat rodent diet (D12102C formula: 10 kcal% fat, 70 kcal% carbohydrates, 20 kcal% protein, 0% cholesterol; Research Diets Inc., New Brunswick, NJ, USA) and assigned as the control group (CON). ApoE^−/−^ mice were placed on a high-fat rodent diet (D12108C: 40 kcal% fat, 40 kcal% carbohydrates, 20 kcal% protein, 1.25% cholesterol; Research Diets) and randomly separated into two groups: high-fat diet (HFD) group and HFD+ YR group whose animals were given 0.75 g/kg body weight YR by daily oral gavage. The animals were cared for in accordance with The Guiding Principles in the Care and Use of Animals. The experiment was approved by the Oil Crops Research Institute Council on Animal Care Committee, Chinese Academy of Agricultural Sciences (Wuhan, China, 2015, IACUC Number: 0016).

### 2.2. Blood Processing and Lipid Analysis

After 10 weeks on the experimental diet, animals were fasted for 12 h. After anesthesia induction with isoflurane, blood was collected from the retro-orbital venous plexus. The serum was obtained by centrifuging at 1500× *g* (10 min, 4 °C) and stored at −80 °C until subsequent analysis. Serum levels of TG, TC, LDL-C, and high-density lipoprotein cholesterol (HDL-C) were determined with commercial kits (Wako Pure Chemical Industries, Tokyo, Japan) by a Hitachi 7020 Chemical Analyzer (Hitachi, Tokyo, Japan). Serum Ox-LDL concentrations were measured with an oxidized LDL ELISA kit (Cloud-Clone, Houston, TX, USA) according to the manufacturer’s instructions.

### 2.3. Atherosclerosis

The aorta from the aortic sinus to the iliac arteries was isolated, stripped of any external fatty deposits, opened longitudinally, and stained with Oil Red O (Sigma, St. Louis, MO, USA). The images were taken with an EPSON GT-X980 photo scanner (Epson, Nagano, Japan). The Oil Red O-stained area and total aortic surface area were quantified with Image-Pro Plus 6.0 software (Media Cybernetics, Rockville, MD, USA). The plaque areas were expressed as the percentage of aorta areas. The aortic roots were embedded with 4% paraformaldehyde, and consecutive sections at a thickness of 10 µm were cut from the aortic sinus to aortic arch. Five sections, selected at 80 µm intervals, were visualized using an Olympus BX61 microscope (Olympus, Tokyo, Japan) after staining with hematoxylin and eosin (H and E). The lesion size was measured using Image Pro Plus 6.0 software (Media Cybernetics, Rockville, MD, USA) from an average of five cross sections. The plaque areas were expressed as the percentage of total areas.

### 2.4. Multiple Cytokine Measurements

Measurement of serum cytokine concentrations were performed with multiplex immunoassay methods using the eBioscience™ ProcartaPlex Mouse Cytokine and Chemokine Convenience Panel 1A (36 plex; Thermo Fisher Scientific, Waltham, MA, USA) following the manufacturer’s instructions.

### 2.5. Statistical Analyses

Results are expressed as mean ± standard error of the mean (SEM). Statistical analysis was performed with the *t*-test or one-way analysis of variance (ANOVA) followed by the least significance difference (LSD) test for post-hoc analysis if the overall differences were significant (*p* < 0.05). All statistical analyses were performed using SPSS 13.0 statistical software (SPSS Inc., Chicago, IL, USA), and *p* values less than 0.05 were considered statistically significant.

## 3. Results

### 3.1. Effects of YR (Yirui) on Atherosclerotic Plaques

**Figure 1:**
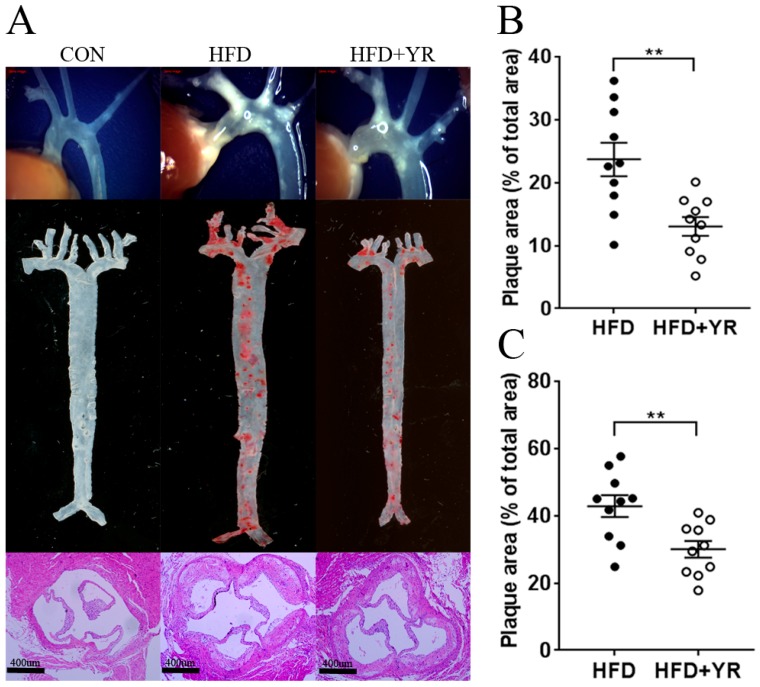
Effects of YR (Yirui) on atherosclerotic lesions in HFD-fed ApoE^−/−^ (high-fat diet-fed apolipoprotein E-deficient) mice. (**A**) Representative photographs of aortic lesions (top), images of Oil Red O-stained (middle), and H and E stained aortic root lesions (bottom); (**B**) Areas of the aortic lesion are expressed as the percentage of aorta areas. Each black filled circle represents an individual ApoE^−/−^ mouse treated with HFD, each open circle represents an individual ApoE^−/−^ mouse treated with HFD+ YR; (**C**) Areas of the aortic sinus lesion are expressed as the percentage of total areas. Each black filled circle represents an individual ApoE^−/−^ mouse treated with HFD, each open circle represents an individual ApoE^−/−^ mouse treated with HFD+ YR. Data are expressed as the mean ± SEM (*n* = 10 animals/group). ** *p* < 0.01 compared to the HFD group.

When the aortic arch was cleaned and all of the surrounding tissues were inspected under a stereomicroscope, no obvious atherogenesis was observed in CON animals (Figure 1A); however, ApoE^−/−^ HFD-fed mice developed severe aortic lesions. Oil Red O-stained aortas and H and E-stained aortic roots showed marked aortic atherosclerotic plaque growth in the HFD group. The enhanced lesion formation was reversed by YR treatment, and quantitative assessment revealed significant reduction in the plaque area of the total aorta (Figure 1B) or aortic root (Figure 1C) in the HFD+ YR group.

### 3.2. Effects of YR (Yirui) on the Serum Lipid Profile

**Figure 2:**
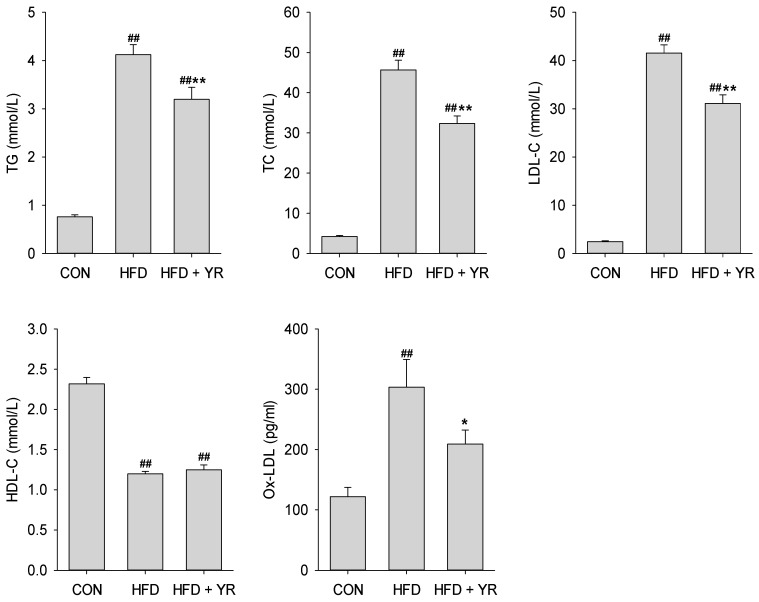
Effects of YR (Yirui) on the serum lipid profile in HFD-fed ApoE^−/−^ (high-fat diet-fed apolipoprotein E-deficient) mice. Bars represent mean ± SEM (*n* = 10 animals/group). ^##^ *p* < 0.01 compared to the CON group, * *p* < 0.05 and ** *p* < 0.01 compared to the HFD group; one-way ANOVA followed by the post-hoc LSD (least significance difference) test.

ApoE^−/−^ mice fed a HFD had greatly increased serum levels of TC, LDL-C, and TG and reduced levels of HDL-C compared with CON animals (Figure 2). YR treatment significantly lowered the levels of TC, LDL-C, and TG, but did not affect HDL-C concentration. The Ox-LDL concentration in HFD-fed ApoE^−/−^ mice was significantly higher than that in CON mice, but YR significantly reduced these levels.

### 3.3. Effects of YR (Yirui) on Cytokine and Chemokine Levels

**Figure 3:**
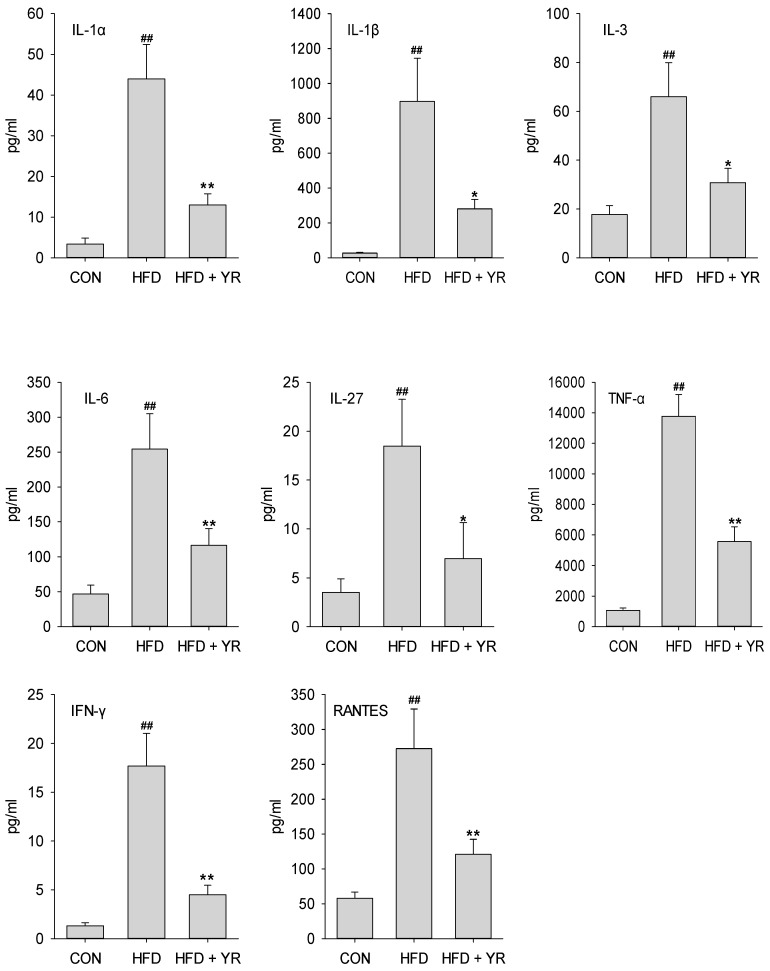
Effects of YR (Yirui) on cytokine and chemokine levels in HFD-fed ApoE^−/−^ (high-fat diet-fed apolipoprotein E-deficient) mice. Bars represent mean ± SEM (*n* = 10 animals/group). ^##^ *p* < 0.01 compared to the CON group, * *p* < 0.05 and ** *p* < 0.01 compared to the HFD group; one-way ANOVA followed by the post-hoc LSD (least significance difference) test.

To determine the systemic effects of YR on the inflammatory response, serum concentrations of inflammatory cytokines and chemokines were evaluated by multi-cytokine analysis. Mice in the HFD group had significantly greater serum levels of interleukin 1 alpha (IL-1α), interleukin-1 beta (IL-1β), interleukin-3 (IL-3), interleukin-6 (IL-6), interleukin-27 (IL-27), tumor necrosis factor alpha (TNF-α), interferon γ (IFN-γ), and regulated on activation, normal T cell expressed and secreted (RANTES) than CON animals (Figure 3); the levels of these inflammatory cytokines were markedly reduced upon YR treatment. The remaining cytokine and chemokine levels were unaffected by YR.

## 4. Discussion

AS is the most common pathological process underlying CVD, which is the leading cause of mortality and morbidity worldwide. To understand its mechanisms, many animal models have been developed; however, there are some differences that have been observed with regard to phenotype and response to treatment. For example, LDL receptor-deficient mice were found to respond better to statins than ApoE^−/−^ mice [30]. Since ApoE^−/−^ mice are the most frequently used mouse models for AS and there is no somato-statin analog in YR capsule components, in this study, we utilized the ApoE^−/−^ mice to explore the protective effects of YR against AS.

In this study, HFD effectively induced the development of AS in ApoE^−/−^ mice, as indicated by the significantly-increased plaque formation in the aorta and aortic sinus. YR led to smaller lesions in the artery and a pronounced reduction in the atherosclerotic plaque burden. Hyperlipidemia, including hypertriglyceridemia and hypercholesterolemia, accelerates the development of AS [31]. Overwhelming evidence has demonstrated that increased TC and LDL-C levels are atherogenic [32,33]. LDL oxidation and the subsequent foam cell formation of macrophages are pivotal events and hallmarks of AS pathogenesis [34]. However, HDL may have cardioprotective effects [32,35] by reversing cholesterol transport and impeding oxidative changes in LDL, protecting the vascular endothelium and exerting anti-inflammatory effects [36,37]. Therefore, the regulation of blood lipid levels, including reducing triglyceride, TC, and LDL-C levels, increasing HDL-C levels, and blocking LDL oxidation, is the most common therapeutic strategy for CVD. Some herbal medicines prevent atherosclerotic lesion development by regulating the lipid profile and inhibiting LDL oxidation [16,38]. In this study, an HFD led to elevated serum levels of TG, TC, and LDL-C, and decreased HDL-C levels. YR treatment notably decreased TG, TC, and LDL-C levels, but did not change HDL-C levels in ApoE^−/−^ mice, resulting in a decrease in the TC/HDL-C and LDL-C/HDL-C ratios [39], thus contributing to cardiovascular protection. Mitigating increased Ox-LDL content in HFD-fed atherogenic animals indicated that blocking LDL oxidation is another therapeutic benefit of YR in AS.

Basic scientific research has highlighted the pivotal role of inflammation in the pathogenesis of AS [13,40], and AS is now recognized as a chronic inflammatory disease [13]. Cytokines are expressed by almost all cells involved in the pathogenesis of AS, and act on various targets to exert multiple effects and participate in all steps of the pathogenesis process [41]. Depending on their effects on AS, cytokines can be broadly classified as pro-atherogenic (e.g., TNF-α, IL-1, and IL-6) and anti-atherogenic (e.g., interleukin-10 (IL-10) and interleukin-35 (IL-35). Therefore, cytokines are key orchestrators of inflammation in AS. In AS, the destructive inflammatory process in the atherosclerotic artery leads to increased levels of pro-inflammatory cytokines and other acute-phase reactants in the blood [40]. However, circulating cytokines, such as IL-1, IL-2, IFN-γ, and TNF-α participate in initiating and sustaining full-blown AS [42]. Thus, anti-inflammatory therapy is a novel direction for treating AS [43]. Consistent with a previous report [44], the development of AS in response to an HFD was accompanied by the substantial production of circulating cytokines, and elevated levels of inflammatory cytokines (e.g., IL-1α, IL-1β, IL-3, IL-6, TNF-α, and IFN-γ) decreased markedly upon YR intervention. Therefore, the anti-inflammatory effects of YR may substantially protect against AS.

In conclusion, YR reduced the atherosclerotic plaque burden, thereby alleviating AS by regulating the lipid profile and exerting anti-inflammatory effects. Since the ingredients of YR are available in China’s health food stores, it may act as a functional food to inhibit AS and provide potential benefits in CVD.
